# New Insights into the Role of NF-κB in Hepatitis B Virus Infection

**DOI:** 10.7150/ijbs.126968

**Published:** 2026-02-11

**Authors:** Shana Halemubieke, Xinru Liu, Ying Yan, Huimin Ji, Huizhen Sun, Jie Ma, Le Chang, Lunan Wang

**Affiliations:** 1 National Center for Clinical Laboratories, Institute of Geriatric Medicine, Chinese Academy of Medical Sciences, Beijing Hospital/National Center of Gerontology, Beijing, P.R. China.; 2 Beijing Engineering Research Center of Laboratory Medicine, Beijing Hospital, Beijing, 100730, P.R. China.; 3 National Center for Clinical Laboratories, Peking Union Medical College, Chinese Academy of Medical Sciences, Beijing, 100730, P.R. China.; 4 Center of Biotherapy, Beijing Hospital, National Center of Gerontology, Institute of Geriatric Medicine, Chinese Academy of Medical Sciences, Beijing, 100730, P. R. China.

**Keywords:** NF-κB, IκB, IKK, hepatitis B virus

## Abstract

The nuclear factor kappaB (NF-κB) is a critical regulator in immune development, responses, and tumorigenesis. A range of signals can stimulate its activation by interacting with the inhibitor of κB (IκB) and IκB kinase (IKK) through the canonical pathway or non-canonical pathway. Activation of NF-κB results in this transcriptional factor binding with specific κB sites of different target genes. The NF-κB signaling pathway is particularly associated with liver diseases including hepatitis B virus (HBV) infection. HBV proteins have been shown to interact with different members of the NF-κB system, either enhancing or suppressing its activity. Here, we summarize current understanding on the interaction between HBV proteins and the NF-κB pathway, along with its association with liver diseases and potential therapeutic targets.

## Introduction

Hepatitis B virus (HBV) infection remains a significant global health concern, affecting more than 250 million chronic carriers and leading to severe liver conditions including cirrhosis and hepatocellular carcinoma (HCC)^1^. It is well-established that HBV significantly influences various cellular signaling pathways and is recognized as the key driver for HCC development^2^. Elucidating the molecular mechanisms underlying HBV pathogenesis is therefore essential for advancing basic research and designing therapeutic interventions.

The NF-κB signaling pathway is critically involved in the control of many physiological and pathological processes. It activates the expression of multiple genes to regulate cell behaviors. Upon activation by diverse stimuli, this signaling cascade initiates downstream target genes^3^. NF-κB activation influences critical biological processes, including immune cell development, inflammation, and stress^4^. Evidence suggests that this signaling pathway has been associated with the pathogenesis of several liver diseases, including hepatitis B and C, alcohol-induced liver inflammation, and HCC^5^.

Here, we briefly outline the NF-κB signaling system and provide a critical assessment of how HBV proteins regulate this pathway. We systematically analyze major controversies in existing studies, examine potential sources of inconsistency, and offer our own insights to clarify these unresolved issues. Beyond HBV infection, we discuss NF-κB's role in other liver diseases and underscore its promise as a therapeutic target, emphasizing recent advances in strategies aimed at modulating NF-κB activation for the treatment of HBV and related liver disorders.

## The NF-κB Pathway

NF-κB is widely acknowledged as a pivotal regulator of various biological processes in innate and adaptive immunity, inflammatory responses, and oncogenesis^6^.

Structurally, the NF-κB family consists of five members (p50, p52, p65/RelA, RelB, and cRel) that form homodimers or heterodimers, with p50 and p52 being derived from the precursor proteins NF-κB1 (p105) and NF-κB2 (p100), respectively^7^. The activity of these various dimers is tightly controlled by inhibitor of κB (IκB) protein. IκB retains NF-κB dimers in the cytoplasm and is phosphorylated and degraded upon activation. The phosphorylation and degradation of IκB are mediated by the IκB kinase (IKK) complex, which consists of two catalytic subunits (IKKα and IKKβ) and the regulatory subunit NEMO^8^.

The NF-κB network consists of canonical and non-canonical (or alternative) pathways with distinct activation mechanisms and biological functions^9^. The canonical pathway responds rapidly to a wide range of stimuli, including inflammatory cytokines, antigen receptors, and pathogen-associated molecules^10^. Engagement of typical ligands, including TNFα, lipopolysaccharides (LPS), and interleukin-1β (IL-1β) with their specific receptors in the cell membrane initiates the recruitment of adaptors (e.g., TGFβ -activated kinase 1 (TAK1)) to the cytoplasmic domain of the receptors^10^. These adaptors facilitate the recruitment of IKK complex via the K63-ubiquitin-binding activity of NEMO, thereby activating IKK complex^11^. Activated IKK complex, primarily through its IKKβ subunit, phosphorylates IκBα at serines 32 and 36, which leads to K48 ubiquitination and degradation by the 26S proteasome of IκBα^11^, ^12^. This permits the nuclear translocation of NF-κB, where it can bind to κB sites to initiate transcription of target genes^3^. This pathway is tightly controlled by an IκB-dependent negative feedback loop to ensure transient activation.

In contrast, the non-canonical pathway is activated only by a limited subset of the tumor necrosis factor (TNF) cytokine family, including B cell-activating factor receptor (BAFF-R), lymphotoxin β receptor (LTβR) or CD40^13^. Under basal conditions, NIK remains undetectable due to constitutive degradation^14^. TNF receptor-associated factor 2 and 3 (TRAF2/3) together with cellular inhibitor of apoptosis protein 1 and 2 (cIAP1/2) promote NIK ubiquitination and subsequent degradation by binding to a specific domain of NIK^15^. Receptor activation induces degradation of TRAF2/3 by cIAP-mediated ubiquitination, which makes it possible for NIK to accumulate and activate IKKα^16^. IKKα subsequently phosphorylates p100, promoting its partial proteolysis into p52, which forms transcriptionally active p52-RelB dimers to translocate to the nucleus^14^. The non-canonical pathway is essential for the establishment and maintenance of primary and secondary lymphoid organs, as well as for coordinating adaptive immune responses^17^.

Overall, the canonical pathway significantly governs cell proliferation, cell survival, angiogenesis, metastasis, inflammation, epithelial-to-mesenchymal transition, and invasion^18^, while the non-canonical pathway provides complementary functions, serving as an auxiliary signaling axis^19^.

## NF-κB in HBV Infection

**Figure 1:**
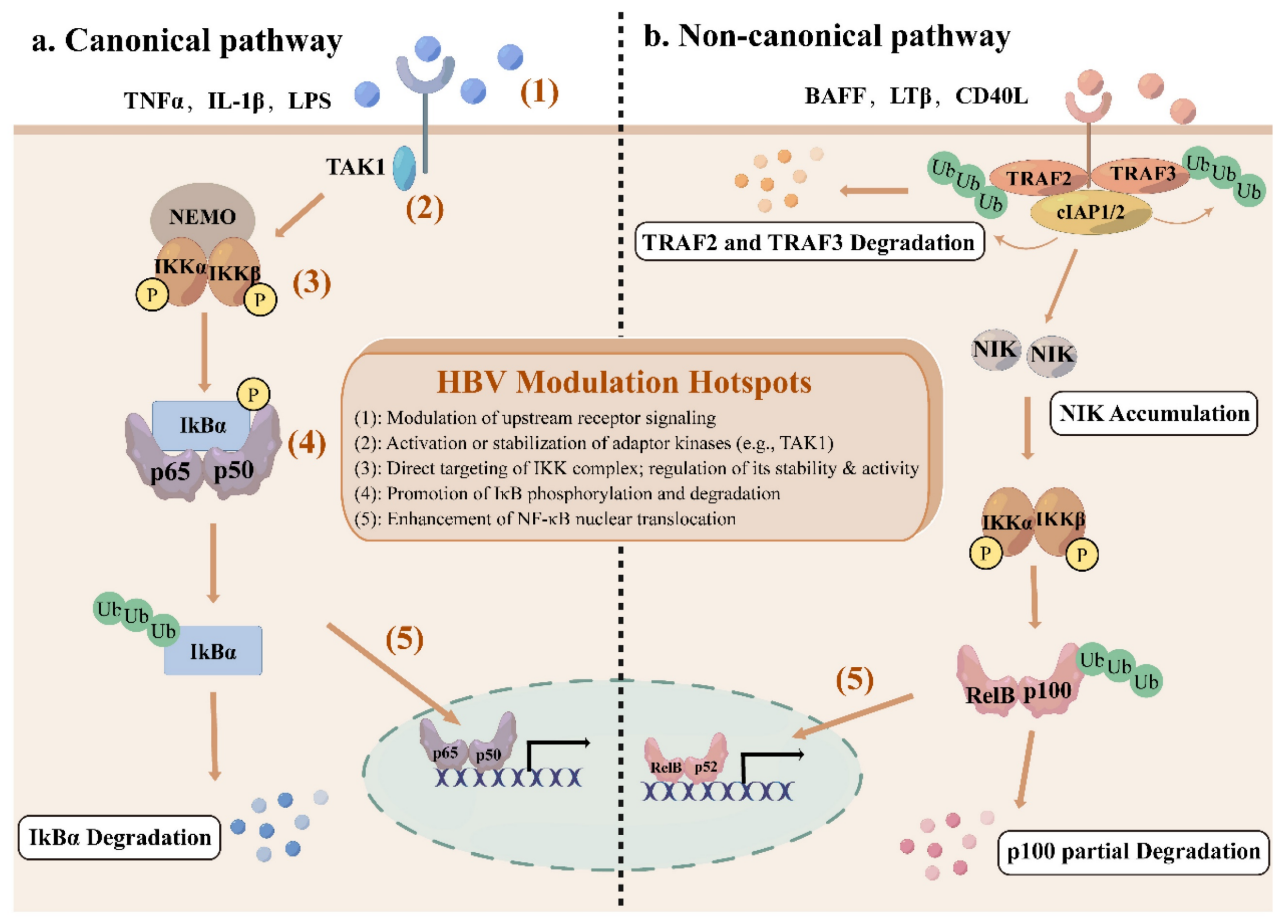
The canonical and non-canonical NF-κB signaling pathway. (a) The canonical NF-κB signaling pathway is activated by cytokines such as TNFα, IL-1β, and LPS, recruiting TAK1 to phosphorylate IKK complex. Activated IKK complex then phosphorylate IκBα, leading to the ubiquitination and degradation of IκBα, and releasing NF-κB subunits to translocate to the nucleus. (b) The non-canonical NF-κB signaling pathway is stimulated by specific members of TNF cytokine family, including BAFF-R, LTβR, and CD40. Receptor activation recruits TRAF2/3, leading to the ubiquitination and degradation of TRAF2/3 by cIAP1/2, resulting in the stabilization and accumulation of NIK. IKKα is phosphorylated by NIK, leading to the ubiquitination and partial degradation of p100 to form p52 and nuclear translocation of p52-RelB dimers. Red numbered hotspots (1)-(5) indicate representative levels at which HBV proteins perturb the pathway, as detailed in subsequent sections and figures.

Hepatitis B virus (HBV) is a partially double-strand DNA virus^20^. Its 3.2kb genome encodes seven proteins, including three different HBsAg-the large (L), medium (M), and small (S) proteins, HBx, HBeAg, HBcAg, and polymerase (Pol)^21^. While the general functions of these proteins are well established, this review focuses on their specific interactions with the NF-κB signaling pathway. During infection, HBV proteins intricately modulate NF-κB by targeting multiple nodes from upstream adaptors and the IKK complex to NF-κB dimers, through direct binding or post-translational modifications such as phosphorylation and ubiquitination. This fine-tuning affects NF-κB nuclear translocation, DNA binding, and target gene expression, enabling the virus to balance immune activation and suppression, which is critical for viral persistence, immune evasion, and hepatocarcinogenesis. The regulatory outcomes are complex and context-dependent, certain HBV proteins can either activate or inhibit NF-κB. Figure 1 integrates the canonical and non-canonical NF-κB pathways with these HBV modulation hotspots, providing a comprehensive overview of this dynamically regulated network in HBV infection. The specific mechanisms by which HBV proteins interact with NF-κB are described below.

### HBsAg: Context-Dependent Dual Regulation of NF-κB Signaling

**Figure 2:**
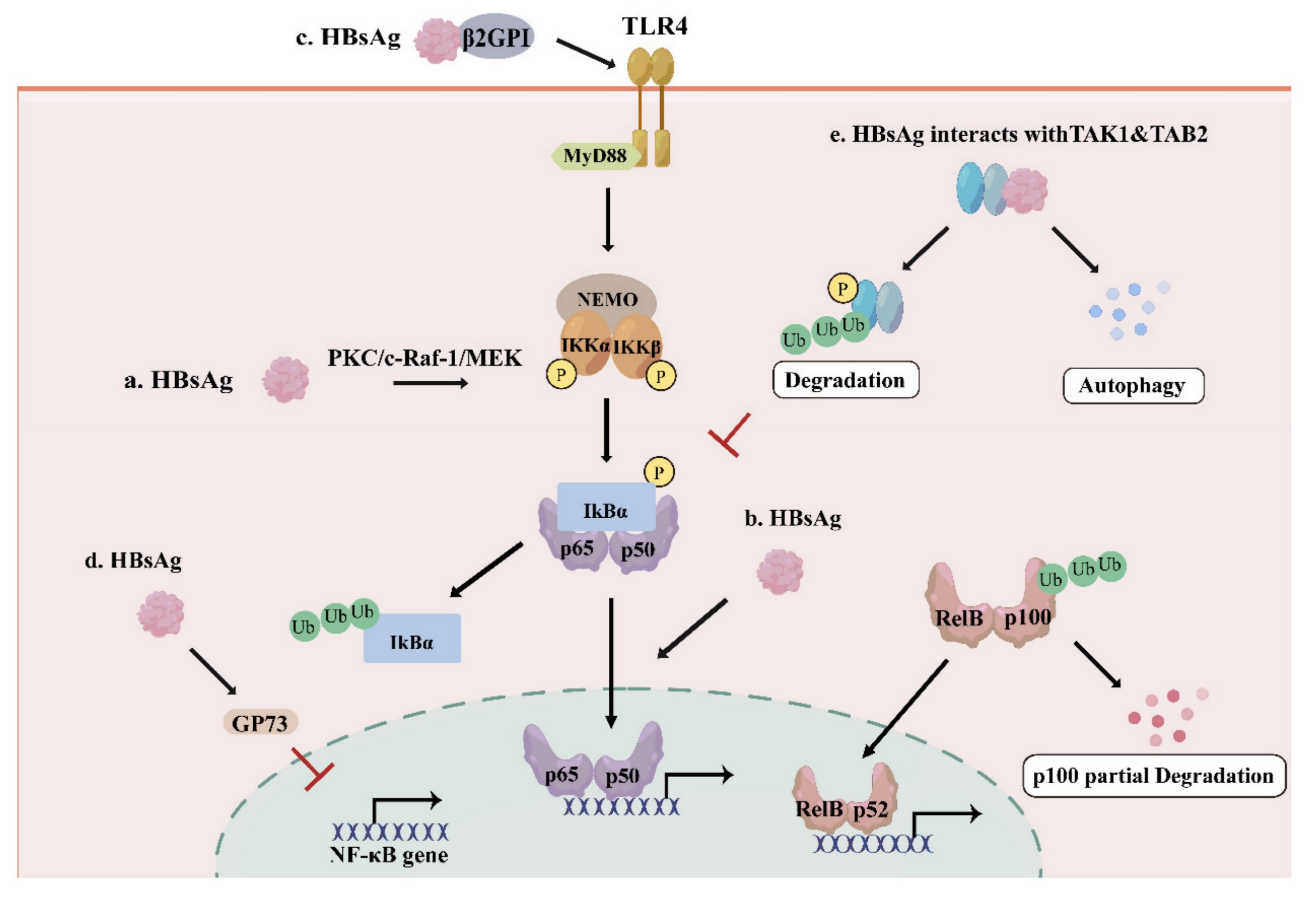
The effect of HBsAg on NF-κB pathway. a. LHBs, MHBs and MHBs^t^ activates NF-κB through PKC/c-Raf-1/MEK axis; b. HBsAg promotes NF-κB nuclear translocation to induce LINC00665, contributing to hepatocarcinogenesis; c. HBsAg/β2GPI activates NF-κB via TLR4/MyD88/IκBα axis; d. HBsAg promotes GP73 expression to repress NF-κB gene expression; e. HBsAg interacts with TAK1 and TAB2 complex to suppress the NF-κB signaling pathway by inhibiting the phosphorylation of TAK1 and promoting autophagic degradation of the complex.

As the viral envelope, HBsAg is also secreted as sub-viral particles which may suppress the host immune system^22^. Large surface proteins (LHBs) include the preS1, preS2, and S domains, middle surface proteins (MHBs) contain the preS2 and S domains, and small surface proteins (SHBs) only consist of the S domain^23^. HBsAg has been extensively implicated in the modulation of the NF-κB signaling, although its effects appear to be highly context-dependent (Figure 2). Several studies demonstrated that C-terminus truncated MHBs (MHBs^t^) and LHBs both can function as transcriptional activators and induce NF-κB activation through protein kinase C (PKC)-dependent activation of the c-Raf-1/mitogen-activated protein kinase kinase (MEK) signal transduction pathway^24^, ^25^ (Figure 2.a). Importantly, transgenic mouse models expressing MHBs(t) in the liver demonstrated sustained PKC/c-Raf-1/Erk2 activation and a higher incidence of liver tumors, supporting a tumor promoter-like role of PreS2 activators and underscoring the pathophysiological relevance of PKC-dependent NF-κB activation in HBV-associated hepatocarcinogenesis. Consistently, MHBs-mediated NF-κB activation promotes the expression and secretion of pro-inflammatory cytokines such as interleukin-6 (IL-6)^26^, supporting a role for HBsAg in inflammatory signaling and hepatocyte proliferation. Moreover, HBsAg-induced nuclear translocation of NF-κB subunits has been linked to the upregulation of the oncogenic long non-coding RNA LINC00665, further implicating HBsAg-driven NF-κB activation in hepatocarcinogenesis^27^ (Figure 2.b). In addition to intracellular signaling, extracellular HBsAg can activate NF-κB through receptor-mediated pathways. HBsAg predominantly interacts with β2-glycoprotein I (β2GPI) via its S domain, forming a complex that triggers NF-κB activation through toll-like receptor 4 (TLR4)/myeloid differentiation factor 88 (MyD88)/IκBα axis by inducing phosphorylation of IκBα at Ser 32/36, revealing the mechanism of HBsAg/β2GPI-activated NF-κB pathway, which may have implications for HBV-induced HCC therapy^28^ (Figure 2.c).

In contrast, accumulating evidence indicates that HBsAg can also suppress NF-κB signaling, thereby facilitating viral persistence. HBsAg-induced upregulation of Golgi protein 73 (GP73) in PBMCs and THP-1 macrophages has been shown to repress NF-κB expression and facilitate HBV replication in HepG2 and Huh7 cells^29^ (Figure 2.d). Another study demonstrates that HBsAg interacts specifically with TAK1 and TAK1-binding protein 2 (TAB2) in vitro and in vivo, as shown by co-immunoprecipitation assays^30^. HBsAg reduces the polyubiquitination of the TAK1-TAB2 complex, suppresses the phosphorylation of TAK1 and IκBα, and even interacts with TAK1 and TAB2 to promote the degradation of the TAK1-TAB2 through autophagy, resulting in the inhibition of the NF-κB pathway^30^ (Figure 2.e). Importantly, consistent inhibitory effects of HBsAg on NF-κB signaling were also observed in liver biopsy specimens from HBV-infected patients and in HBV-infected HepG2-hNTCP cells^30^, underscoring the physiological relevance of this mechanism. These findings suggest that HBsAg-mediated suppression of NF-κB may contribute to innate immune evasion and facilitate HBV replication during chronic infection.

HBsAg demonstrates context-dependent dual regulation of the NF-κB signaling. It activates NF-κB primarily in models of hepatocyte transformation and carcinogenesis, where pro-inflammatory and pro-survival signals may favor viral replication or drive proliferation. Conversely, HBsAg suppresses NF-κB in chronic infection models such as in patient liver biopsies and HBV-infected cell lines. This inhibitory capacity directly weakens the host's innate immune response and is a key mechanism for viral immune evasion and the maintenance of persistent infection. Thus, the direction of HBsAg regulation on NF-κB may primarily depend on cell type, stage of infection, and molecular form of HBsAg.

### HBx: Multifaceted Activation of NF-κB Signaling

**Figure 3:**
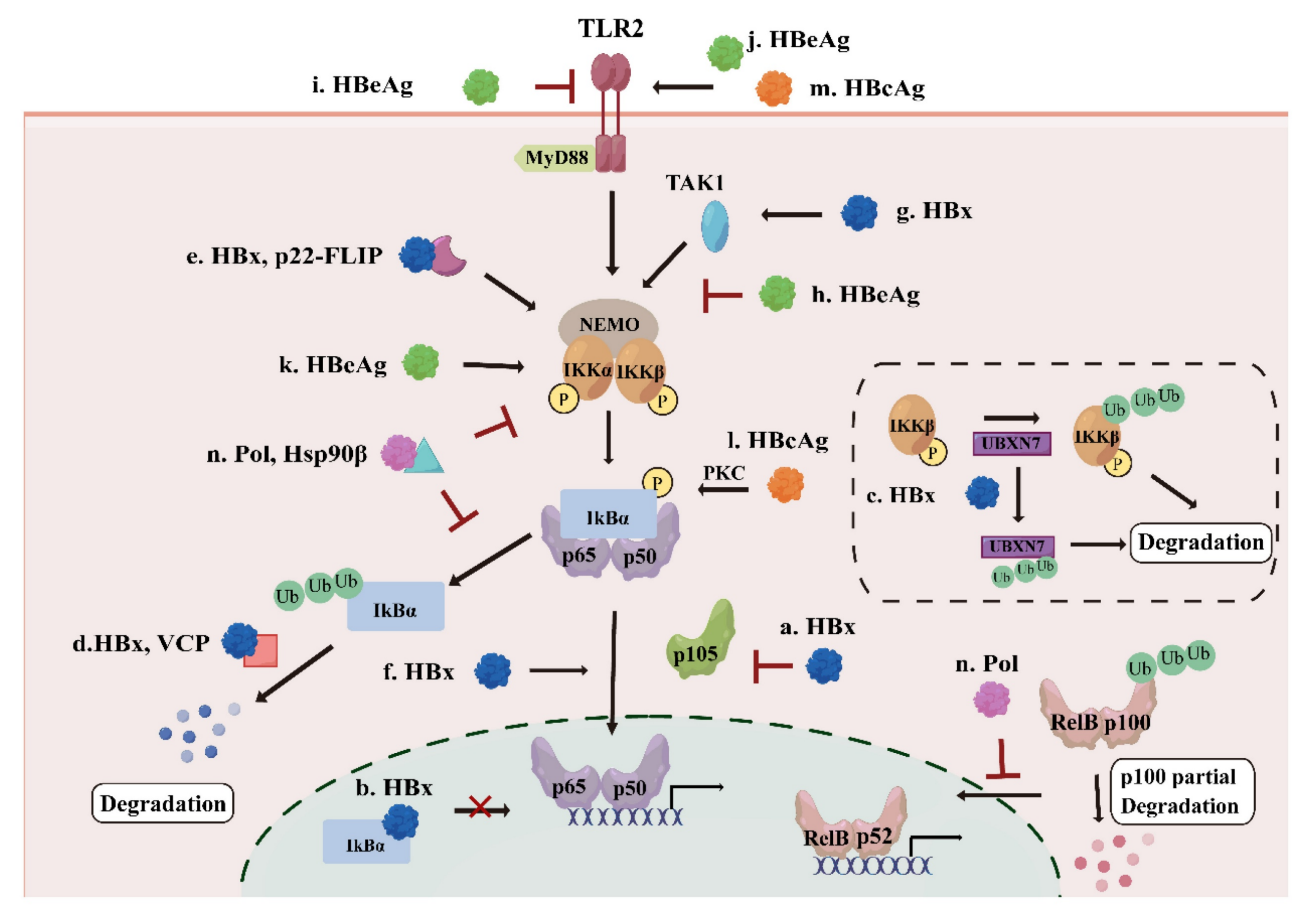
The effect of other HBV proteins on NF-κB pathway. a. HBx induces phosphorylation of IκBα and decrease of p105 to activate the NF-κB pathway; b. HBx prevents nuclear IκBα from reassociating with DNA-bound NF-κB; c. HBx induces the degradation UNXN7 to stimulate the NF-κB pathway; d. HBx interacts with VCP to accelerate the degradation of IκBα, leading to the activation of the NF-κB pathway; e. HBx forms a ternary complex with p22-FLIP and NEMO to stimulate the NF-κB pathway; f. HBx interacts with p65 and promotes NF-κB subunits nuclear translocation; g. HBx activates the NF-κB pathway through up-regulation of TAK1; h. HBeAg suppresses the NF-κB activation by suppressing TAK1-NEMO interaction and decreasing the ubiquitination of NEMO; i/j. HBeAg activates or inhibits the NF-κB pathway through TLR2; k. HBeAg increases the phosphorylation of IKKα to stimulate the NF-κB pathway; l. HBcAg stimulates the PKC/NF-κB signaling pathway; m. HBcAg activates the NF-κB pathway through TLR2; n. HBV Pol interacts with Hsp90β to inhibit the phosphorylation of IKK complex, suppressing the NF-κB pathway, HBV Pol can also inhibit nuclear translocation of NF-κB dimers in the non-canonical pathway.

HBx is essential for initiating and maintaining HBV replication after infection^31^. It modulates many signaling pathways associated with inflammation and cell proliferation^32^. NF-κB was among the first HBx-responsive motifs identified, and HBx has been shown to interact with NF-κB signaling components through multiple mechanisms^33^. At the upstream level, HBx promotes NF-κB activation primarily by targeting the inhibitory machinery. HBx induces phosphorylation and destabilization of IκBα, and reduces cytoplasmic p105 levels, thereby promoting the release and nuclear accumulation of RelA-containing NF-κB dimers^34^ (Figure 3.a). Research has also indicated that HBx can directly interact with IκBα to facilitate NF-κB nuclear translocation and may prevent nuclear IκBα from reassociating with DNA-bound NF-κB^35^ (Figure 3.b). In addition, HBx enhances IKK complex activity by inducing degradation of the NF-κB inhibitor UBXN7, stabilizing IKKβ to stimulate the NF-κB pathway and promote virus replication^36^ (Figure 3.c). HBx not only interacts directly with the NF-κB pathway, but also influences NF-κB activity through other proteins. For instance, HBx associates with valosin-containing protein (VCP), which accelerates the degradation of ubiquitinated IκBα, and enhances the VCP-mediated activation of NF-κB^37^ (Figure 3.d). A cleavage product of the cellular anti-apoptotic protein (c-FLIP), p22-FLIP, forms a ternary complex with HBx and NEMO and synergistically enhances HBx-induced NF-κB activation^38^ (Figure 3.e). Once the NF-κB pathway is activated directly or indirectly, HBx activates oncogenic pathways that induce proliferation, inflammation, and tumorigenesis in HBV infection^39^.

Beyond direct modulation of NF-κB inhibitory components, HBx also amplifies NF-κB signaling at the transcriptional level. Evidence indicates that HBx could upregulate p65 expression by binding to the p65 promoter, as well as promoting the phosphorylation of p65 and facilitating its nuclear translocation^40^ (Figure 3.f). Elevation of TBK1 expression has also been observed in a HBx-expressing cell line and HBV-positive serum from HCC tissue samples, indicating that HBx may contribute to HCC development by activating the NF-κB pathway^41^ (Figure 3.g). Collectively, these findings indicate that HBx acts as a potent and multifaceted activator of the canonical NF-κB signaling.

Functionally, HBx-mediated NF-κB activation orchestrates a broad transcriptional program that contributes to inflammation, immune modulation, and hepatocellular carcinoma progression. In HBV-infected patients, HBx-mediated NF-κB activation induces the expression of inflammatory cytokines and immune regulators, including TNF-α, MIG, IL-23, and the decoy receptor DcR3, which correlate with inflammation, liver injury, and viral load^42^-^45^. Moreover, NF-κB-dependent upregulation of gp96 enhances viral production^46^, while induction of SHP2, S100A9, and oncogenic microRNAs such as miR-1269b promotes fibrosis, tumor cell proliferation, migration, and invasion during HCC development^47^-^49^.

Despite the predominance of evidence supporting NF-κB activation by HBx, a limited number of studies report inhibitory effects under specific conditions. For instance, a study carried out in 2019 found that overexpression of HBx suppressed NF-κB phosphorylation in normal hepatocytes, inhibiting the expression of high-mobility group protein box1 (HMGB1)^50^. This apparent discrepancy may reflect differences in cellular context, suggesting that HBx-mediated regulation of NF-κB may shift from activation to suppression depending on the host cell type. Taken together, HBx predominantly promotes inflammation and hepatocarcinogenesis by activating the NF-κB pathway in hepatoma cells, whereas it may also suppress NF-κB in normal hepatocytes.

### HBeAg: Conditional Bidirectional Modulation of NF-κB Signaling

Clinical observations indicate that HBeAg is pivotal in HBV chronicity and the modulation of immune responses^51^. HBeAg interferes with host signaling and dampens immune detection, which not only shields infected hepatocytes from apoptosis but also fosters a microenvironment favorable for hepatocellular carcinoma development^52^. Mechanistically, HBeAg predominantly suppresses NF-κB activation in hepatocytes and immune cells. HBeAg can associate with NEMO and inhibit its K63-linked ubiquitination, thereby disrupting TAK1-NEMO interaction and IKK complex phosphorylation, leading to reduced transcription of NF-κB-dependent proinflammatory cytokines such as IL-6 and IL-8 and attenuation of IL-1β-mediated NF-κB activation^53^ (Figure 3.h). Additionally, HBeAg suppresses TLR2, which normally triggers the NF-κB pathway to induce the expression of cytokines, including IL-1, IL-6 and TNF-α^54^ (Figure 3.i). Moreover, HBeAg inhibits NF-κB dependent priming of the NOD-like receptor (NLR)-family pyrin domain-containing 3 (NLRP3) inflammasome, further dampening innate immune responses^55^. These inhibitory effects collectively support immune tolerance and contribute to the establishment and maintenance of chronic HBV infection.

Notably, accumulating evidence suggests that HBeAg-mediated regulation of NF-κB is not uniformly suppressive but is highly dependent on cellular context and antigen processing. In macrophages, HBeAg has been shown to activate the TLR2/NF-κB pathway, leading to macrophage activation and subsequent promotion of hepatic stellate cells (HSCs) proliferation and contraction, thereby contributing to liver fibrosis^56^ (Figure 3.j). Consistently, both HBsAg and HBeAg could upregulate the triggering receptor expressed on myeloid cells-1 (TREM-1) pathway through NF-κB in monocytes, amplifying inflammatory responses associated with liver fibrosis^57^. Furthermore, according to a recent study published in 2023, the cleaved form of HBeAg, p22, can enhance the phosphorylation of IKKα to activate the NF-κB pathway and suppress TNFα-induced apoptosis^58^ (Figure 3.k). This highlights that post-translational processing of HBeAg can convert it from an immune suppressor into a pro-survival and pro-inflammatory factor. Taken together, HBeAg exerts bidirectional modulation of the NF-κB pathway, acting primarily as a suppressor in hepatocytes to foster immune tolerance, while also activating the pathway in myeloid cells or in the cleaved form to drive fibrotic inflammation.

### HBcAg: Predominant Activation of NF-κB Signaling

HBcAg has various effects on hepatoma cell migration and proliferation, apoptosis pathways, metabolic regulation, immune responses, and epigenetic and genetic events^59^-^61^. In contrast to HBeAg, HBcAg more consistently acts as an activator of the NF-κB pathway across multiple cell types. In hepatocytes, HBcAg increases the IL-6 expression and secretion via activating NF-κB^62^. In dendritic cells (DCs), HBcAg dose-dependently promotes proliferation and inhibits apoptosis through the protein kinase C (PKC)/NF-κB pathway, accompanied by upregulation of the NF-κB downstream anti-apoptotic protein Bcl-2^63^ (Figure 3.l). Similarly, in macrophages, HBcAg enhances the activation of the NF-κB pathway through TLR2 recognition^64^ (Figure 3.m). These findings suggest that HBcAg serves as a potent immune stimulus that promotes inflammatory signaling and cell survival, which may contribute to chronic inflammation and liver disease progression.

### HBV Pol: Dual Suppression of Canonical and Non-Canonical NF-κB Signaling

HBV Pol is responsible for the replication of HBV^65^. HBV Pol performs diverse functions, including DNA- or RNA-dependent DNA polymerization, pregenome RNA (pgRNA) packaging, protein priming for reverse transcription initiation, and RNase activity^66^. Beyond its canonical role in viral replication, accumulating evidence indicates that HBV Pol also functions as a potent modulator of host innate immune signaling, particularly targeting the NF-κB pathway.

HBV Pol has been shown to suppress NF-κB activation in a dose-dependent manner across multiple cell types, suggesting that immune modulation is an intrinsic and conserved function of this viral protein. Mechanistically, HBV Pol interferes with both canonical and non-canonical NF-κB signaling pathways. In the non-canonical pathway, HBV Pol blocks the nuclear translocation of NF-κB, while in the canonical pathway, HBV Pol interacts with heat shock protein 90β (Hsp90β), leading to impaired phosphorylation of IKKα/β and inhibition of IκBα degradation, thereby resulting in the suppression of NF-κB-mediated transcription of downstream genes, such as IL-6 and IL-8^67^ (Figure 3.n). In vitro experiments on HepAD38, HepG2, and HepG2.215 cells further demonstrate that HBV Pol is indispensable for the inhibitory effects of HBV on NF-κB signaling, underscoring its central role in viral immune evasion^68^. From a biological perspective, the ability of HBV Pol to simultaneously suppress both rapid, inflammation-driven canonical NF-κB signaling and the more sustained non-canonical pathway highlights a strategic viral mechanism to dampen host innate immune responses during active replication. By restraining NF-κB-mediated cytokine production and antiviral signaling, HBV Pol likely facilitates pgRNA reverse transcription and viral genome amplification while minimizing immune-mediated clearance.

As described above, HBV executes a masterful strategy to balance the host NF-κB pathway through the coordinated, bidirectional manipulations of its encoded proteins. A comprehensive overview of NF-κB modulation by HBsAg, HBx, HBeAg, HBcAg, and HBV Pol is provided in Table 1.

### NF-κB as a Host Restrictive Factor in HBV Infection

While many proteins of HBV modulate NF-κB, NF-κB can also regulate HBV infection. Apolipoprotein B enzyme catalytic subunit 3B (APOBEC3B, A3B) has been suggested to function as an antiviral enzyme following a long LTβR agonization, the miR-138-5p decreases and fails to decay A3B mRNA, and then activated NF-κB signaling induces strong A3B mRNA expression, allowing elimination of nuclear HBV cccDNA by A3B^69^, ^70^. Entry of polyguluronate sulfate (PGS) into HepG2.2.15 cells has been shown to potentially trigger the NF-κB signaling pathway, leading to an upregulated interferon response that restricts HBV translation^71^. Hepatocyte nuclear factor 1α (HNF1α), a key regulator of hepatocyte metabolism and proliferation, suppresses HBV replication and gene expression by promoting p65 expression and protein stability^72^.

Collectively, these observations suggest that NF-κB restricts HBV infection through coordinated actions on cccDNA stability, viral gene expression, and host transcriptional regulation. Such antiviral functions provide a strong selective pressure for HBV to evolve multiple strategies to suppress NF-κB signaling, thereby reinforcing the central role of the NF-κB pathway in the host-virus arms race during chronic HBV infection.

## NF-κB in Other Liver Diseases

The NF-κB signaling pathway is closely associated with other various liver diseases, including liver fibrosis, liver cirrhosis, and HCC^73^. It exhibits distinct functions in liver physiology and pathology. It is reported that NF-κB can protect the liver from TNF-α-induced apoptosis^73^. While protecting hepatocytes from cell death, NF-κB initiates appropriate inflammatory and immune responses; however, both insufficient and excessive activation of NF-κB may have a negative effect on the liver^5^. In response to liver injury, Kupffer cells show strong activation of NF-κB, leading to the secretion of proinflammatory cytokines, including the TNF-α and IL-6 which act as promoters of fibrosis and HCC^74^. In the process of liver fibrosis, HSCs are activated rapidly after stimulation and transdifferentiate into fibrogenic and proliferative myofibroblast-like cells^75^. Activated HSCs exhibit apoptosis resistance via NF-κB signaling pathway, which upregulates anti-apoptotic proteins including Bcl-2; besides, NF-κB also plays a critical role in the secretion of inflammatory chemokines in HSCs, including C-C motif chemokine ligand 3 (CCL3) and C-X-C motif chemokine ligand 2 (CXCL2), and also increases the infiltration of inflammatory cells to the liver, resulting in a positive feedback on HSCs activation^76^. In different HCC models, activation of NF-κB appears to function contradictorily. In a model of inflammation-driven HCC, NF-κB is regarded as a tumor-promoting factor because it protects transformed hepatocytes from programmed cell death (PCD); conversely, NF-κB is also suggested to suppress diethylnitrosamine (DEN)-induced HCC because of increased hepatocarcinogenesis after specific deletion of IKKβ in hepatocytes^77^. Furthermore, in HBsAg-driven HCC model, inhibition of the canonical NF-κB signaling pathway leads to the disruption of compensatory liver regeneration, a down-regulation of the unfolded protein response (UPR) regulator 78-kDa glucose-regulated protein and enhanced ER stress which promotes DNA damage and increases HCC incidence^78^.

## Potential Therapeutic Targets in the NF-κB Pathway

Given the pivotal role of the NF-κB pathway in HBV infection, inflammation, and hepatocarcinogenesis, targeting this pathway has emerged as a promising yet complex strategy for the treatment of HBV-related liver disease.

A wide range of therapeutic agents have been shown to interfere with the NF-κB signaling through distinct mechanisms, including IKK inhibitors (Aspirin, BAY 11-7821), monoclonal antibodies (anti-PD-1/PD-L1, anti-IL-1), proteasome inhibitors (Bortezomib, Carfilzomib), inhibitors of NF-κB nuclear translocation and DNA binding, as well as tyrosine kinase inhibitors^79^. These agents not only modulate inflammatory and immune responses but may also impact HBV replication and the development of hepatocellular carcinoma (HCC) by disrupting HBV-host interactions mediated through NF-κB.

For instance, HBx upregulates TRAIL-R3 through the NF-κB pathway, thereby inhibiting both TRAIL-induced apoptosis and TRAIL-mediated suppression of HBV replication, while this mechanism can be disrupted by the NF-κB inhibitor BAY 11-7085^80^. In addition, activation of NF-κB signaling during HBV infection is associated with elevated JMJD2D expression, which facilitates HBx stability and promotes cccDNA transcription. Therefore, inhibition of the NF-κB pathway may represent a potential strategy to suppress JMJD2D expression and reduce HBV cccDNA transcriptional activity^81^. Moreover, NKILA, a long noncoding RNA which interacts with NF-κB, attenuates HBx-induced NF-κB activation by interfering with the HBx-p65 interaction, and may act as a negative regulator of HBV replication^82^. In the context of HCC, aspirin has demonstrated the ability to induce ferroptosis by suppressing NF-κB-mediated transcription of SLC7A11, thereby inhibiting tumor growth^83^. Together, these findings highlight the therapeutic potential of NF-κB pathway inhibition across multiple stages of HBV pathogenesis and HBV-associated liver disease. Notably, several pharmacological agents that indirectly or directly modulate NF-κB signaling have already been approved for clinical use in some chronic diseases. These include anti-TNF-α monoclonal antibodies, such as infliximab and adalimumab, which are widely used in rheumatoid arthritis^84^, ^85^; proteasome inhibitors, including bortezomib, which suppress NF-κB activation by preventing IκB degradation, are used in multiple myeloma^86^. Despite their clinical success in autoimmune diseases and hematological malignancies, these agents have not been directly applied to the treatment of chronic HBV infection or HBV-associated hepatocellular carcinoma. The limited clinical translation of NF-κB targeting therapies in HBV-related liver diseases highlights the need to move beyond global pathway suppression toward more precise and context-dependent modulation strategies. Systemic inhibition of NF-κB signaling carries a substantial risk of immunosuppression and increased susceptibility to infections, which is particularly concerning in patients with chronic viral infection. In addition, tumor cells may bypass NF-κB blockade through compensatory signaling pathways, thereby limiting therapeutic efficacy. Furthermore, certain NF-κB modulating agents, such as IKKβ inhibitors ML120B and BMS-345542, have been associated with hepatotoxicity in clinical trials^86^, raising additional safety concerns in patients with underlying liver disease.

In this regard, future therapeutic approaches should account for cell type, stage of HBV infection, and specific components of the NF-κB signaling cascade. For example, in hepatocytes, NF-κB activation may promote viral replication, suggesting that selective inhibition of hepatocyte NF-κB during the establishment or chronic phases of infection could reduce viral persistence. In contrast, in immune cells, NF-κB activity is critical for mounting effective antiviral responses, and thus enhancing or preserving the NF-κB pathway in these cells may improve immune-mediated clearance of infected hepatocytes. NF-κB regulation may also need to be dynamically adjusted depending on the infection stage, as early activation may help initiate innate and adaptive immunity, whereas sustained or excessive activation during chronic infection may contribute to inflammation, fibrosis, and hepatocarcinogenesis. A cell-type and stage-specific approach to modulating NF-κB could thus enable interventions that simultaneously suppress viral replication and limit liver pathology. Additionally, rational combination strategies that integrate NF-κB modulators with existing antivirals may hold synergistic potential. While current nucleos(t)ide analogues effectively suppress viral DNA synthesis, their combination with careful NF-κB modulation may help restore immune function and promote clearance of infected cells, potentially translating these mechanistic insights into clinically feasible regimens aimed at functional cure.

In conclusion, although caution is warranted due to the risk of immune impairment, the precise and strategic modulation of the NF-κB pathway represents a valuable and promising adjunctive approach in the evolving therapeutic landscape against HBV.

## Summary and Prospects

The NF-κB signaling pathway is of great importance in the regulation of inflammation, immune responses, and cancer. The interactions between HBV and the NF-κB signaling pathway are complicated. HBV proteins can modulate the NF-κB signaling pathway either directly or indirectly, often with the help of other proteins, particularly affecting the canonical pathway, and thereby leading to the activation or inhibition of target genes transcription. Notably, certain HBV proteins exert context-dependent and even opposing effects on NF-κB signaling under different cellular or immunological conditions. To date, most studies have focused on HBV interaction with the canonical pathway, while the interplay between the non-canonical NF-κB pathway and HBV remains largely unknown. Addressing this knowledge gap represents an important direction for future research, as the non-canonical pathway may contribute to immune regulation, viral persistence, and liver disease progression in ways that are mechanistically distinct from canonical signaling.

Importantly, a deeper understanding of HBV and NF-κB crosstalk is expected to have significant translational implications. While NF-κB represents a promising therapeutic target, its essential role in antiviral immunity poses substantial challenges for clinical intervention. Future studies should therefore aim to delineate context-specific and cell-type-specific functions of NF-κB signaling during different stages of HBV infection, with an emphasis on identifying regulatory nodes that can be precisely modulated. Such insights may facilitate the development of targeted or combination therapeutic strategies that complement existing antiviral treatments and contribute to the long-term goal of achieving a functional cure for HBV infection.

## Figures and Tables

**Table 1:** Influences of HBV proteins on NF-κB signaling pathway

HBV proteins	Interacting proteins	Influences on NF-κB signaling pathway	Target genes
HBsAg	β2GPI^28^	Activation: IKK^87^, NF-κB subunits^27^	TREM-1^57^, LINC00665^27^
	GP73^29^, TAK1-TAB2^30^	Suppression: IκBα^30^	
HBx	TAK1^41^, VCP^37^, c-FLIP^38^, UBXN7	Activation: IκBα^34^, ^35^, ^37^, IKK (IKKβ, NEMO)^36^, ^38^, NF-κB subunits^39^, ^40^	gp96^46^, IL-23^44^, DcR3^45^, TNF-α^42^, SHP2^47^, S100A9^48^, miR-1269b^88^, MIG^43^, HMGB1^50^
	-	Suppression^50^	
HBeAg	-	Activation: through TLR2/NF-κB pathway^54^, IKKα^58^	TREM-1^57^, IL-6/ IL-8^53^
	-	Suppression: IKK (NEMO)^53^	
HBcAg	-	Activation: through PKC/NF-κB pathway^63^	IL-6^62^, Bcl-2^63^
Polymerase	Hsβ90^67^	Suppression: IKK^67^, RelB-p100^67^	IL-6/IL-8^67^
